# Using machine learning algorithms to enhance IoT system security

**DOI:** 10.1038/s41598-024-62861-y

**Published:** 2024-05-27

**Authors:** Hosam El-Sofany, Samir A. El-Seoud, Omar H. Karam, Belgacem Bouallegue

**Affiliations:** 1 College of Computer Science, King Khalid University, Abha, Kingdom of Saudi Arabia; 2 Faculty of Informatics and Computer Science, British University in Egypt-BUE, Cairo, Egypt; 3 Electronics and Micro-Electronics Laboratory (E. μ. E. L), Faculty of Sciences of Monastir, University of Monastir, Monastir, Tunisia

**Keywords:** Internet of Things, Sustainable development goals, Sustainable cities and communities, IoT security, Machine learning, Computer science, Information technology

## Abstract

The term “Internet of Things” (IoT) refers to a system of networked computing devices that may work and communicate with one another without direct human intervention. It is one of the most exciting areas of computing nowadays, with its applications in multiple sectors like cities, homes, wearable equipment, critical infrastructure, hospitals, and transportation. The security issues surrounding IoT devices increase as they expand. To address these issues, this study presents a novel model for enhancing the security of IoT systems using machine learning (ML) classifiers. The proposed approach analyzes recent technologies, security, intelligent solutions, and vulnerabilities in ML IoT-based intelligent systems as an essential technology to improve IoT security. The study illustrates the benefits and limitations of applying ML in an IoT environment and provides a security model based on ML that manages autonomously the rising number of security issues related to the IoT domain. The paper proposes an ML-based security model that autonomously handles the growing number of security issues associated with the IoT domain. This research made a significant contribution by developing a cyberattack detection solution for IoT devices using ML. The study used seven ML algorithms to identify the most accurate classifiers for their AI-based reaction agent’s implementation phase, which can identify attack activities and patterns in networks connected to the IoT. The study used seven ML algorithms to identify the most accurate classifiers for their AI-based reaction agent’s implementation phase, which can identify attack activities and patterns in networks connected to the IoT. Compared to previous research, the proposed approach achieved a 99.9% accuracy, a 99.8% detection average, a 99.9 F1 score, and a perfect AUC score of 1. The study highlights that the proposed approach outperforms earlier machine learning-based models in terms of both execution speed and accuracy. The study illustrates that the suggested approach outperforms previous machine learning-based models in both execution time and accuracy.

## Introduction

Technology such as cloud computing, cloud edge, and software-defined networking (SDN) have significantly increased users’ reliance on their infrastructure. Consequently, the number of threats faced by these users has also risen. As a result, security management during IoT system development has become increasingly difficult and complex. The IoT can be described as an electrical network that connects physical objects, such as sensors, with software that makes it possible for them to exchange, examine, and gather data. Various sectors use IoT applications, including the military, personal healthcare, household appliances, and agriculture production infrastructure^1^. This research attempts to achieve the Sustainable Cities and Communities Goal (SDG 11) included in the UN Sustainable Development Goals (SDG)^2^. Addressing the challenges and finding solutions for the IoT require considering a wide range of factors. It is crucial for solutions to encompass the entire system to provide comprehensive security. However, most IoT devices operate without human interaction, making them susceptible to unauthorized access. Therefore, it is imperative to enhance the existing security techniques to safeguard the IoT environment^3^. ML techniques can offer potential alternatives for securing IoT systems, including:

- *Intrusion detection and prevention* ML can create IoT intrusion detection and prevention (IDPS) tools. ML algorithms can analyze network traffic, device logs, and other data related to known attacks or suspicious activity.
- *Anomaly detection* ML algorithms can learn IoT device behavior and network interactions through anomaly detection. ML models can detect unusual IoT activity using real-time data. This helps detect security breaches like unauthorized access or malicious acts and prompt appropriate responses.
- *Threat intelligence and prediction* ML can analyze big security data sets and provide insights. ML models may discover new risks, anticipate attack pathways, and give actionable insight to IoT security practitioners by analyzing data from security feeds, vulnerability databases, and public forums.
- *Firmware and software vulnerability analysis* Researchers may use ML to analyze IoT firmware and software for vulnerabilities. ML models may discover IoT device firmware and software security problems by training on known vulnerabilities and coding patterns. This helps manufacturers repair vulnerabilities before deployment or deliver security patches quickly.
- *Behavior-based authentication* ML algorithms can learn IoT devices and user behavior. By analyzing device usage patterns, ML models may create predictable behavior profiles. ML can require extra authentication or warn for illegal access when a device or user deviates considerably from the learned profile.
- *Data privacy and encryption* ML can assist in ensuring data privacy and security in IoT systems. ML algorithms may provide homomorphic encryption, which permits calculations on encrypted data. ML can perform data anonymization and de-identification to safeguard sensitive data and facilitate analysis and insights.

In general, ML techniques must be used in conjunction with other security measures to offer complete security for IoT systems. ML algorithms and methods have been applied in various tasks, including machine translation, regression, clustering, transcription, detection, classification, probability mass function, sampling, and estimation of probability density. Numerous applications utilize ML techniques and algorithms, such as spam identification, image and video recognition, customer segmentation, sentiment analysis, demand forecasting, virtual personal assistants, detection of fraudulent transactions, automation of customer service, authentication, malware detection, and speech recognition^4^.

In addition, IoT and ML integration can enhance the devices of IoT levels of security, thereby increasing their reliability and accessibility. ML’s advanced data exploration methods play an important role in elevating IoT security from only providing security for communication devices to intelligent systems with a high level of security^5^.

ML-based models have emerged as a response to cyberattacks within the IoT ecosystem, and the combination of Deep Learning (DL) and ML approaches represents a novel and significant development that requires careful consideration. Numerous uses, including wearable smart gadgets, smart homes, healthcare, and Vehicular Area Networks (VANET), necessitate the implementation of robust security measures to safeguard user privacy and personal information. The successful utilization of IoT is evident across multiple sectors of modern life^6^. By 2025, we expect that the IoT will have an economic effect of $2.70–$6.20 trillion. Research findings indicate that ML and DL techniques are key drivers of automation in knowledge work, thereby contributing to the economic impact. There have been many recent technological advancements that are shaping our world in significant ways. By 2025, we expect an estimated $5.2–$6.7 trillion in annual economic effects from knowledge labor automation^7^.

This research study addresses the vulnerabilities in IoT systems by presenting a novel ML-based security model. The proposed approach aims to address the increasing security concerns associated with the Internet of Things. The study analyzes recent technologies, security, intelligent solutions, and vulnerabilities in IoT-based smart systems that utilize ML as a crucial technology to enhance IoT security. The paper provides a detailed analysis of using ML technologies to improve IoT systems’ security and highlights the benefits and limitations of applying ML in an IoT environment. When compared to current ML-based models, the proposed approach outperforms them in both accuracy and execution time, making it an ideal option for improving the security of IoT systems. The creation of a novel ML-based security model, which can enhance the effectiveness of cybersecurity systems and IoT infrastructure, is the contribution of the study. The proposed model can keep threat knowledge databases up to date, analyze network traffic, and protect IoT systems from newly detected attacks by drawing on prior knowledge of cyber threats.

The study comprises five sections: “Related works” section presents a summary of some previous research. “IoT, security, and ML” section introduces the Internet of Things’ security and ML aspects. “The proposed IoT framework architecture” section presents the proposed IoT framework architecture, providing detailed information and focusing on its performance evaluation. “Result evaluation and discussion” section provides an evaluation of the outcomes and compares them with other similar systems. We achieve this by utilizing appropriate datasets, methodologies, and classifiers. “Conclusions and upcoming work” section concludes the discussion and outlines future research directions.

## Related works

The idea of security in IoT devices has been recently articulated in studies that analyze the security needs at several layers of architecture, such as the application, cloud, network, data, and physical layers. Layers have examined potential vulnerabilities and attacks against IoT devices, classified IoT attacks, and explained layer-based security requirements^8^. On the other hand, industrial IoT (IIoT) networks are vulnerable to cyberattacks. Developing IDS is important to secure IIoT networks. The authors presented three DL models, LSTM, CNN, and a hybrid, to identify IIoT network breaches^9^. The researchers used the UNSW-NB15 and X-IIoTID datasets to identify normal and abnormal data, then compared them to other research using multi-class, and binary classification. The hybrid LSTM + CNN model has the greatest intrusion detection accuracy in both datasets. The researchers also assessed the implemented models’ accuracy in detecting attack types in the datasets^9^.

In Ref.^10^, the authors introduced the hybrid synchronous-asynchronous privacy-preserving federated technique. The federated paradigm eliminates FL-enabled NG-IoT setup issues and protects all its pieces with Two-Trapdoor Homomorphic Encryption. The server protocol blocks irregular users. The asynchronous hybrid LEGATO algorithm reduces user dropout. By sharing data, they assist data-poor consumers. In the presented model, security analysis ensures federated correctness, auditing, and PP. Their performance evaluation showed higher functionality, accuracy, and reduced system overheads than peer efforts. For medical devices, the authors of Ref.^11^ developed an auditable privacy-preserving federated learning (AP2FL) method. By utilizing Trusted Execution Environments (TEEs), AP2FL reduces issues about data leakage during training and aggregation activities on both servers and clients. The authors of this study aggregated user updates and found data similarities for non-IID data using Active Personalized Federated Learning (ActPerFL) and Batch Normalization (BN).

In Ref.^12^, the authors addressed two major consumer IoT threat detection issues. First, the authors addressed FL’s unfixed issue: stringent client validation. They solved this using quantum-centric registration and authentication, ensuring strict client validation in FL. FL client model weight protection is the second problem. They suggested adding additive homomorphic encryption to their model to protect FL participants’ privacy without sacrificing computational speed. This technique obtained an average accuracy of 94.93% on the N-baIoT dataset and 91.93% on the Edge-IIoTset dataset, demonstrating consistent and resilient performance across varied client settings.

Utilizing a semi-deep learning approach, SteelEye was created in Ref.^13^ to precisely detect and assign responsibility for cyberattacks that occur at the application layer in industrial control systems. The proposed model uses category boosting and a diverse range of variables to provide precise cyber-attack detection and attack attribution. SteelEye demonstrated superior performance in terms of accuracy, precision, recall, and Fl-score compared to state-of-the-art cyber-attack detection and attribution systems.

In Ref.^14^, researchers developed a fuzzy DL model, an enhanced adaptive neuro-fuzzy inference system (ANFIS), fuzzy matching (FM), and a fuzzy control system to detect network risks. Our fuzzy DL finds robust nonlinear aggregation using the fuzzy Choquet integral. Metaheuristics optimized ANFIS attack detection’s error function. FM verifies transactions to detect blockchain fraud and boost efficiency. The first safe, intelligent fuzzy blockchain architecture, which evaluates IoT security threats and uncertainties, enables blockchain layer decision-making and transaction approval. Tests show that the blockchain layer’s throughput and latency can reveal threats to blockchain and IoT. Recall, accuracy, precision, and F1-score are important for the intelligent fuzzy layer. In blockchain-based IoT networks, the FCS model for threat detection was also shown to be reliable.

In Ref.^15^, the study examined Federated Learning (FL) privacy measurement to determine its efficacy in securing sensitive data during AI and ML model training. While FL promises to safeguard privacy during model training, its proper implementation is crucial. Evaluation of FL privacy measurement metrics and methodologies can identify gaps in existing systems and suggest novel privacy enhancement strategies. Thus, FL needs full research on “privacy measurement and metrics” to thrive. The survey critically assessed FL privacy measurement found research gaps, and suggested further study. The research also included a case study that assessed privacy methods in an FL situation. The research concluded with a plan to improve FL privacy via quantum computing and trusted execution environments.

## IoT, security, and ML

### IoT attacks and security vulnerabilities

**Figure 1:**
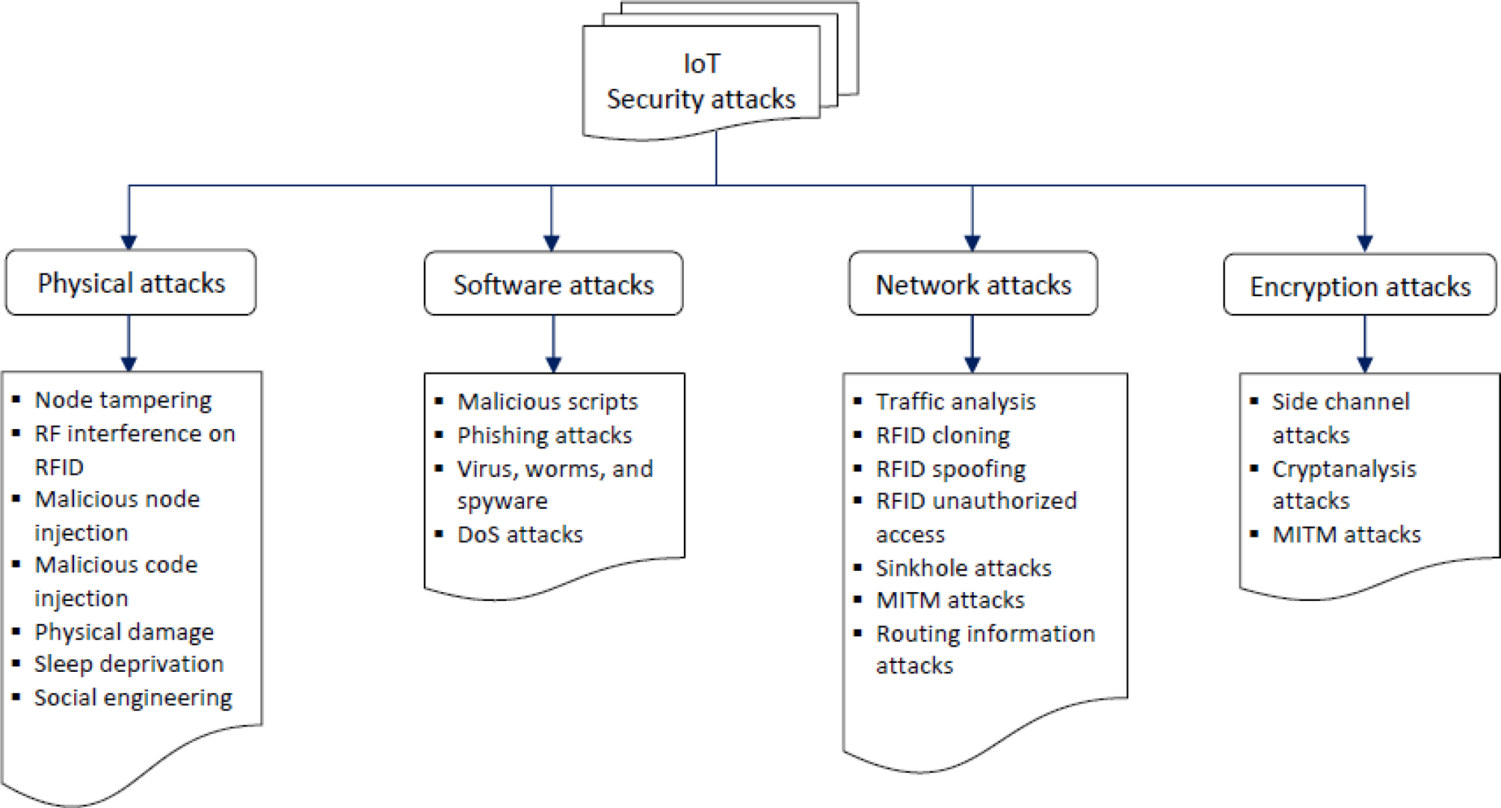
Types of IoT attacks.

Critical obstacles standing in the way of future attempts to see IoT fully accepted in society are security flaws and vulnerabilities. Everyday IoT operations are successfully managed by security concerns. In contrast, they have a centralized structure that results in several vulnerable points that may be attacked. For example, unpatched vulnerabilities in IoT devices are a security concern due to outdated software and manual updates. Weak authentication in IoT devices is a significant issue due to easy-to-identify passwords. Attackers commonly target vulnerable Application Programming Interfaces (APIs) in IoT devices using code injections, a man-in-the-middle (MiTM), and Distributed Denial-of-Service (DDoS)^16^. Unpatched IoT devices pose risks to users, including data theft and physical harm. IoT devices store sensitive data, making them vulnerable to theft. In the medical field, weak security in devices such as heart monitors and pacemakers can impede medical treatment. Figure 1 illustrates the types of IoT attacks (threats)^17^. Unsecured IoT devices can be taken over and used in botnets, leading to cyberattacks such as DDoS, spam, and phishing. The Mirai software in 2016 encouraged criminals to develop extensive botnets for IoT devices, leading to unprecedented attacks. Malware can easily exploit weak security safeguards in IoT devices^18^. Because there are so many connected devices, it may be difficult to ensure IoT device security. Users must follow fundamental security practices, such as changing default passwords and prohibiting unauthorized remote access^19^. Manufacturers and vendors must invest in securing IoT tool managers by proactively notifying users about outdated software, enforcing strong password management, disabling remote access for unnecessary functions, establishing strict API access control, and protecting command-and-control (C&C) servers from attacks.

### IoT applications’ support security issues

Security is a major requirement for almost all IoT applications. IoT applications are expanding quickly and have impacted current industries. Even though operators supported some applications with the current technologies of networks, others required greater security support from the IoT-based technologies they use^20^. The IoT has several uses, including home automation and smart buildings and cities. Security measures can enhance home security, but unauthorized users may damage the owner’s property. Smart applications can threaten people’s privacy, even if they are meant to raise their standard of living. Governments are encouraging the creation of intelligent cities, but the safety of citizens’ personal information may be at risk^21,22^.

Retail extensively uses the IoT to improve warehouse restocking and create smart shopping applications. Augmented reality applications enable offline retailers to try online shopping. However, security issues have plagued IoT apps implemented by retail businesses, leading to financial losses for both clients and companies. Hackers may access IoT apps to provide false details regarding goods and steal personal information^23^. Smart agriculture techniques include selective irrigation, soil hydration monitoring, and temperature and moisture regulation. Smart technologies can result in larger crops and prevent the growth of mold and other contaminants. IoT apps monitor farm animals’ activity and health, but compromised agriculture applications can lead to the theft of animals and damage to crops. Intelligent grids and automated metering use smart meters to monitor and record storage tanks, improve solar system performance, and track water pressure. However, smart meters are more susceptible to cyber and physical threats than traditional meters. Advanced Metering Infrastructure (AMI) connects all electrical appliances in a house to smart meters, enabling communication and security networks to monitor consumption and costs. Adversary incursions into such systems might change the data obtained, costing consumers or service providers money^24^. IoT apps in security and emergency sectors limit access to restricted areas and identify harmful gas leaks. Security measures protect confidential information and sensitive products. However, compromised security in IoT apps can have disastrous consequences, such as criminals accessing banned areas or erroneous radiation level alerts leading to serious illnesses^25^.

### IoT security attacks based on each layer

**Figure 2:**
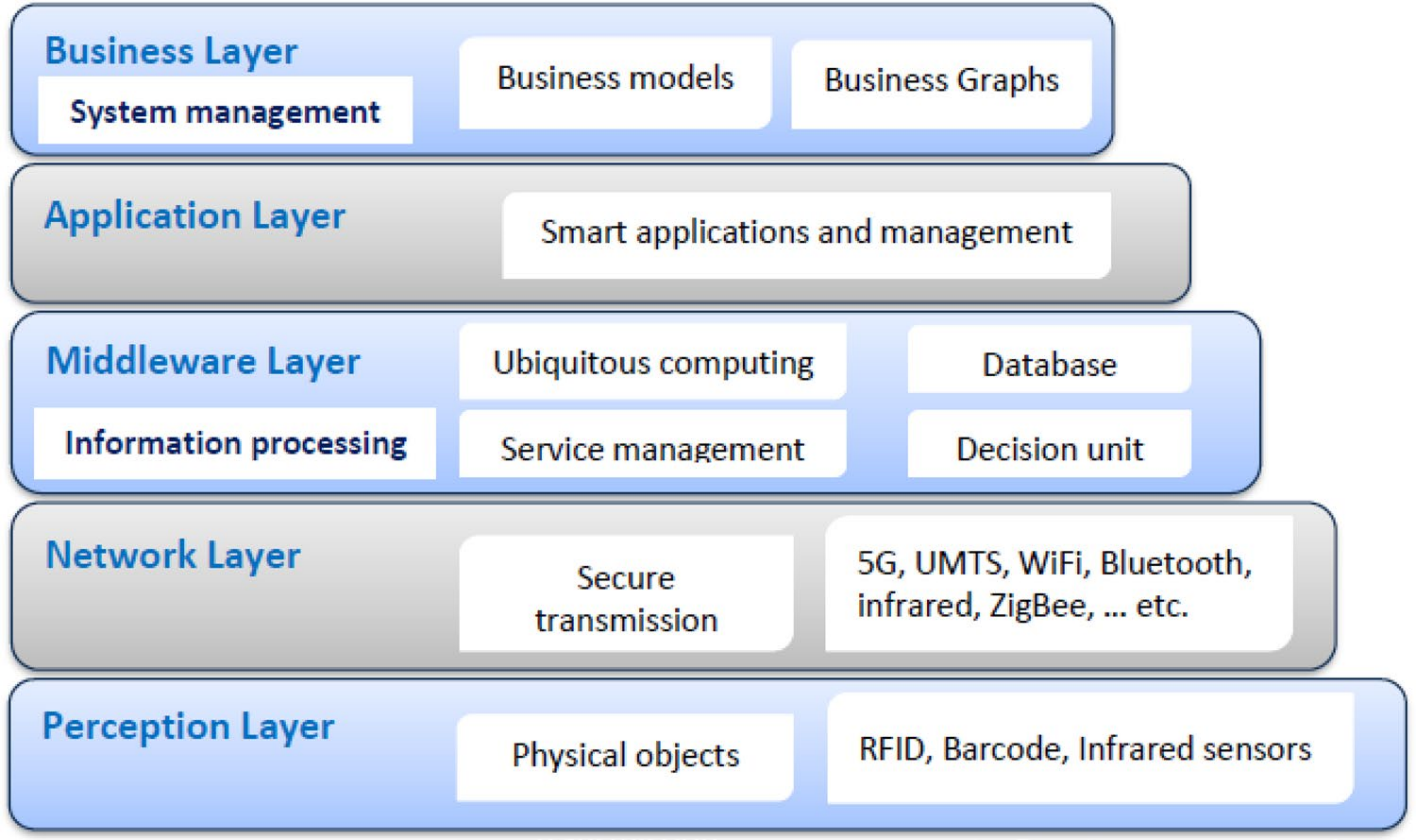
IoT ecosystem five-layer architecture.

IoT devices’ architecture includes five layers: perception, network Layer, middleware (information processing), application, and business (system management). Figure 2 illustrates how the development of IoT ecosystems has changed from a three-layer to a five-layer approach. IoT threats can be physical or cyber, with cyberattacks being passive or active. IoT devices can be physically damaged by attacks, and various IoT security attacks based on each tier are described^26^. Perception layer attacks are intrusions on IoT physical components, for example, devices and sensors. Some of the typical perception layer attacks are as follows:

1. *Botnets* Devices get infected by malware called botnets, like Mirai. The bot’s main objectives are to infect improperly configured devices and assault a target server when given the order by a botmaster^27^.
2. *Sleep deprivation attack* Attacks from sleep deprivation are linked to battery-powered sensor nodes and equipment. Keeping the machines and devices awake for a long time is the aim of the sleep disturbances assault^28^.
3. *Node tampering and jamming* Node tampering attacks are launched by querying the machines to acquire accessibility to and change confidential data, like routing data tables and cryptographic shared keys. A node jamming assault, on the other hand, occurs when perpetrators breach the radio frequencies of wireless sensor nodes^29^.
4. *Eavesdropping* By allowing the attacker to hear the information being transferred across a private channel, eavesdropping is an exploit that puts the secrecy of a message in danger^30^.

These attacks can harm most or all IoT system physical components and can be prevented by implementing appropriate security measures.

Network layer attacks aim to interfere with the IoT space’s network components, which include routers, bridges, and others. The following are some examples of network layer attacks:

- *Man-in-the-middle (MiTM)* This threat involves an attacker posing as a part of the communication networks and directly connecting to another user device^31^.
- *Denial of service (DoS)* Attackers who use DoS techniques generate numerous pointless requests, making it challenging for the user to access and utilize IoT gadgets.
- *Routing attacks* Malicious nodes engage in routing-type assaults to block routing functionality or to perform DoS activities.
- *Middleware attacks* An assault on middleware directly targets the IoT system’s middleware components. Cloud-based attacks, breaches of authentication, and signature packaging attacks are the three most common forms of middleware attacks.

These attacks can be prevented by implementing appropriate security measures.

Smart cities, smart grids, and smart homes are some examples of apps included in the application layer. An application layer attack relates to the security flaws in IoT apps. Here are a few examples of application layer attacks^32^:

- *Malware* The use of executable software by attackers to interfere with network equipment is known as malware.
- *Phishing attack* This is a sort of breach that seeks to get users’ usernames and passwords by making them appear to be reliable entities.
- *Code injection attack* The main goal of an injector attack into a program or script code is to inject an executable code into the memory space of the breached process.

Appropriate security measures can help prevent these attacks as well.

### Overview of ML within the IoT

IoT systems are susceptible to hackers because they lack clear boundaries and new devices are always being introduced. There is a possibility to create algorithms that can learn about the behavior of objects and other IoT components inside such large networks by utilizing ML and DL approaches. By using these techniques to predict a system’s expected behavior based on past experiences, security protocols can be developed to a significant extent.

#### ML techniques and their applications in IoT

ML techniques play an essential role in analyzing and extracting insights from the massive amount of data produced by IoT devices. Here are some popular ML techniques and their applications in the IoT:

1. *Supervised learning* This type of algorithm learns from labeled training data. Various applications in the IoT can utilize supervised learning, such as:
  *Anomaly detection* By training ML models to recognize abnormal patterns or behaviors in IoT sensor data, we can identify anomalies or potential security breaches.
  *Predictive maintenance* By analyzing past sensor data, supervised learning algorithms can predict equipment failures or maintenance requirements. This enables the implementation of proactive maintenance measures, leading to a decrease in downtime.
  *Environmental monitoring* ML models can learn from sensor data to predict environmental conditions like air quality, water pollution, or weather patterns.
2. *Unsupervised learning* Unsupervised learning algorithms extract patterns or structures from unlabeled data without predefined categories. In IoT, unsupervised learning techniques find applications such as:
  *Clustering* ML models can group similar IoT devices or data points, facilitating resource allocation, load balancing, or identifying network segments.
  *Dimensionality reduction* Unsupervised learning techniques like autoencoders or principal component analysis (PCA) make it easier to analyze IoT data.
  *Behavioral profiling* Unsupervised learning can help in understanding the normal behavior of IoT devices or users, enabling the detection of deviations or anomalies.
3. *Reinforcement learning* Reinforcement learning aims to maximize a reward by training an agent how to interact with its environment and use feedback to improve its performance. The following applications use reinforcement learning on the IoT.
  *Energy management* ML models can learn optimal energy allocation strategies for IoT devices to maximize energy efficiency or minimize costs.
  *Adaptive IoT systems* Reinforcement learning can be used to optimize IoT system parameters or configurations based on real-time feedback and changing conditions.
  *Smart resource allocation* ML models can learn to allocate resources dynamically based on demand, user preferences, or changing network conditions.
4. *Deep learning* DL algorithms, especially deep neural networks, excel at processing complex data and extracting high-level features. In IoT, DL has various applications, including:
  *Image and video analysis* DL models can analyze images or video streams from IoT devices, enabling applications like object detection, surveillance, or facial recognition.
  *Natural language processing (NLP)* DL techniques can process and understand text or voice data from IoT devices, enabling voice assistants, sentiment analysis, or chatbots.
  *Time-series analysis* DL models, such as long short-term memory (LSTM) or recurrent neural networks (RNNs) networks, can analyze time-series sensor datasets for predicting future values or detecting anomalies.

#### ML for IoT security

ML is a promising approach for defending IoT devices against cyberattacks. It offers a unique strategy for thwarting assaults and provides several benefits, including designing sensor-dependent systems, providing real-time evaluation, boosting security, reducing the flowing data, and utilizing the large quantity of data on the Internet for all individualized user applications. The influence of ML on the IoT’s development is crucial for enhancing practical smart applications. ML has garnered scientific attention recently and is being applied to IoT security as well as the growth of numerous other industries. Effective data exploration methods for identifying “abnormal” and “normal” IoT components and behavior of devices inside the IoT ecosystem are DL and ML. Consequently, to transform the security of IoT systems from enabling secure Device-to-Device (D2D) connectivity to delivering intelligence security-based systems, ML/DL techniques are needed^33^.

#### Enhancing IoT security using the algorithms of ML

ML approaches, such as ensemble learning, k-means clustering, Random Forest (RF), Association Rule (AR), Decision Tree (DT), AdaBoost, Support Vector Machine (SVM), XGBoost, and K-Nearest Neighbor (KNN), have benefits, drawbacks, and applications in IoT security. DT, a natural ML technique, resembles a tree, with branches and leaves that serve as nodes in the model. In classification, SVM maximizes the distance between the closest points and the hyperplane to classify the class^34^. In identifying DDoS attacks, RF performs better than SVM, ANN, and KNN. A Principal Component Analysis (PCA) with KNN and classifier softmax has been suggested in Ref.^35^ to develop a system that has great time efficiency while still having cheap computation, which enables it to be employed in IoT real-time situations.

#### Limitations of applying ML in networks of IoT

Using ML approaches for IoT networks has limitations because of dedicated processing power and IoT machines’ limited energy. IoT networks generate data with a variety of structures, forms, and meanings, and traditional ML algorithms are ill-equipped to handle these massive, continuous streams of real-time data. The semantic and syntactic variability in this data is evident, particularly in the case of huge data, and heterogeneous datasets with unique features pose problems for effective and uniform generalization. ML assumes that all the dataset’s statistical attributes are constant, and the data must first go through preprocessing and cleaning before fitting into a particular model. However, in the real world, data comes from multiple nodes and has different representations with variant formatting, which presents challenges for ML algorithms^36^.

## The proposed IoT framework architecture

### Fundamental concepts and methodologies

- *Software defined networking (SDN) *SDN is a cutting-edge networking model that separates the data plane from the control plane. This improves network programmability, adaptability, and management, and it also enables external applications to control how the network behaves. The SDN’s three basic components are communication interfaces, controllers, and switches. Cognitive judgments were imposed on the switches by a central authority (i.e., the SDN controller). It keeps the state of the system up to date by changing the flow rules of the appropriate switches. IoT systems’ success and viability depend on SDN adoption. To handle IoT networks’ huge data flows and minimize bottlenecks, SDN’s routing traffic intelligence and improving usage of the network are essential. This connection may be applied at many layers in the IoT network, including enabling end-to-end IoT traffic control, core, access, and cloud networks (where creation, processing, and providing of data takes place). SDN also enhances IoT security, for example, tenant traffic isolation, tracking centralized security based on the network’s global view, and dropping of traffic at the edge of the network to ward off malignant traffic.
- *Network function virtualization (NFV)* Virtualization in network contexts is called *network function virtualization* (NFV). NFV separates software from hardware, adding value and reducing capital and operational costs. The European Telecommunications Standards Institute (ETSI) has standardized this approach’s novel design for use in telecommunications systems. The architecture of ETSI NFV has three basic components:
  - *Virtualization infrastructure* Virtualization technologies are found in this layer in addition to needed hardware that offers abstractions to resources for Virtualized Network Functions (VNFs). Cloud platforms handle networking, data processing, and storage.
  - *Virtual network functions* VNFs replace specific hardware equipment for network functions. They scale and cost-effectively handle network services across numerous settings.
  - *Management and orchestration* Block of Management and orchestration (MANO) is a component of ETSI NFV and is responsible for communicating with the VNF layer and the infrastructure layer. It manages monitoring VNFs, configuration, instantiation, and global resource allocation.
  The ecosystem of the IoT is given value by virtualized resources of the network, explaining its variability and quick expansion. NFV and SDN can offer advanced virtual monitoring tools like Deep Packet Inspectors (DPIs) and Intrusion Detection Systems (IDSs). They can provide scalable network security equipment, as well as deploy and configure on-demand components, such as authentication systems and firewalls, to defend against attacks that have been identified by monitoring agents. When processing for security is offloaded from resource-constrained IoT devices to virtual instances, the resulting boost in efficiency and drop in energy consumption clear the way for other useful applications to be implemented. IoT security hardware lacks NFV’s flexibility and enhanced security. NFV’s value-added features improved IoT security, even if they did not replace current solutions.

- *Machine learning (ML) *ML is an algorithmic artificial intelligence (AI) discipline that uses techniques to give intelligence to devices and computers. ML methods include *unsupervised*, *supervised*, and *reinforcement* learning. They are typically used in the security of networks. ML is used to specify and precisely identify the security regulations of the data plane. In mitigating a sort of attack given by tagging traffic networks or creating policies to access control, the difficulty is to fine-tune key security protocol parameters. Moreover, several ML approaches may prevent IoT attacks.
  - *Supervised learning* In algorithms of supervised learning, the model output is known even though the underlying relationships between the data are unknown. This model is often trained with two datasets: One for “testing” and “evaluating” the driven model and another to “learn” from. Within the context of security, it is common to compare a suspected attack to a database of known threats.
  - *Unsupervised learning* Data is not pre-labeled, and the model is unknown. It sets it apart from supervised learning. It aims to classify and find patterns in the data.
  - *Reinforcement learning* It looks at problems and methods to enhance its model through study. It employs trial and error and incentive mechanisms to train its models in a novel way. A metric known as the “value function” is determined by tracking the output’s success and applying the reward to its formula. This value tells the model how well it is evaluated, so it may adjust its behavior accordingly.

### The proposed security model

**Figure 3:**
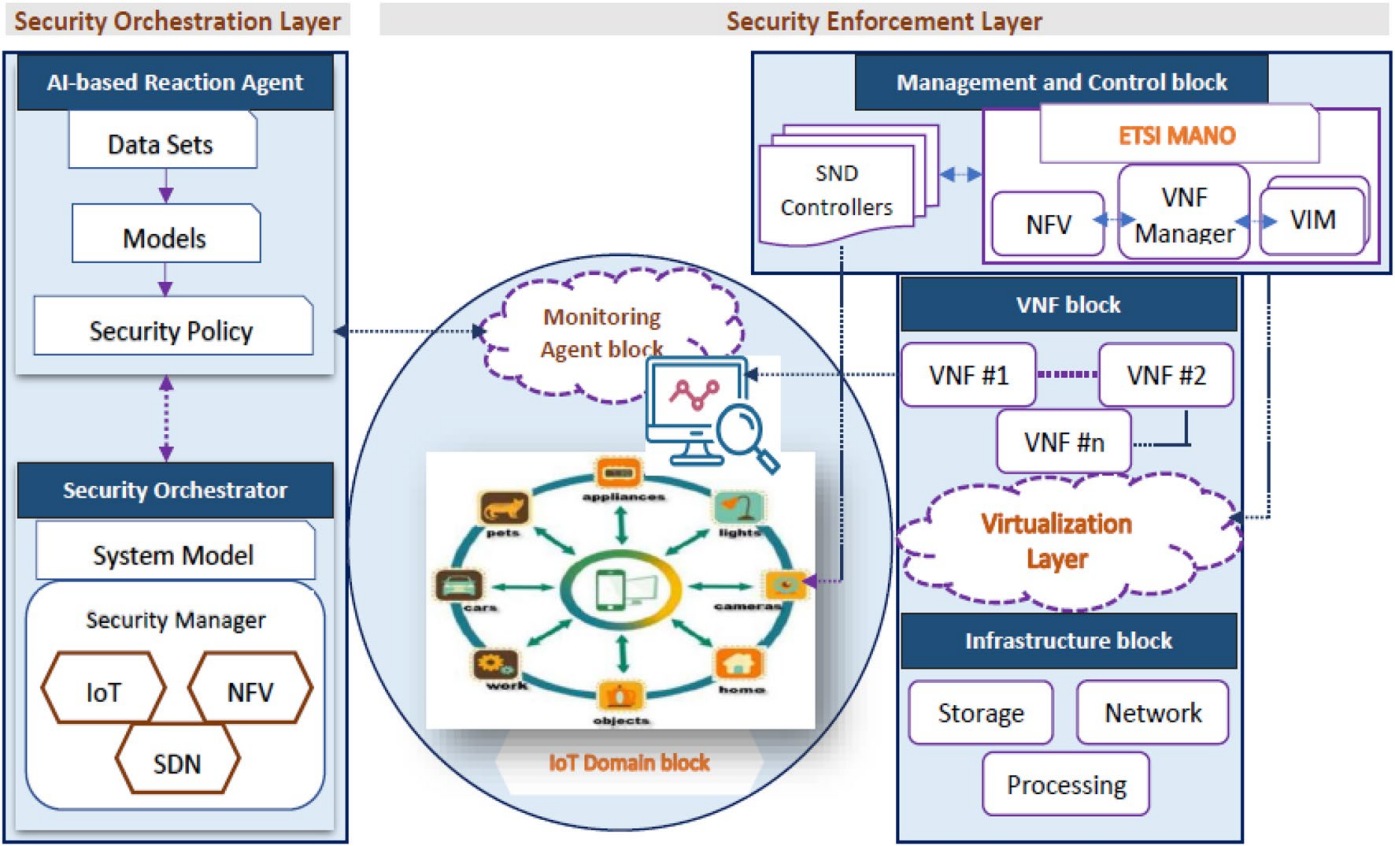
The proposed ML-based security model.

**Figure 4:**
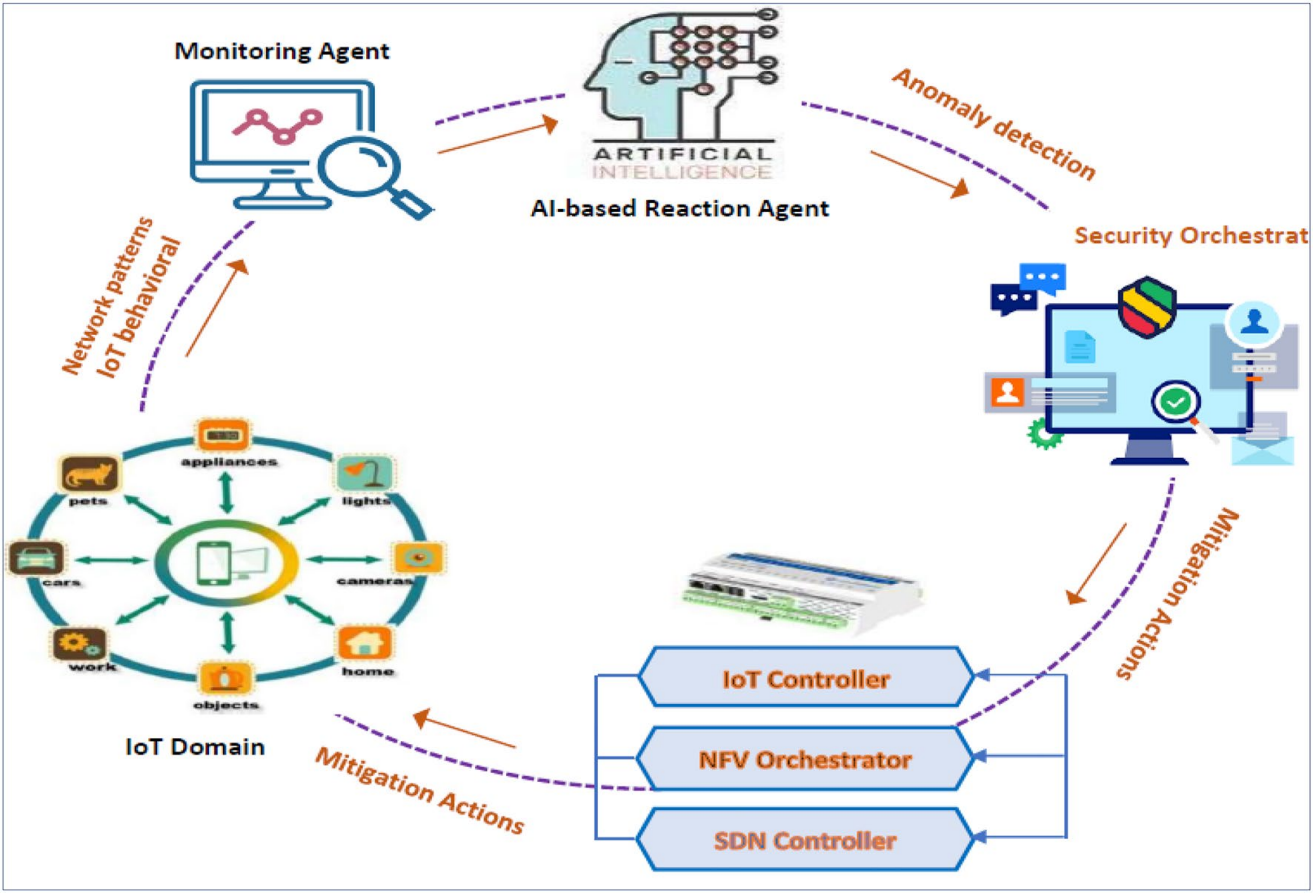
Automation with a closed loop, from detection to prevention.

Figure 3 illustrates the proposed ML-based security model to address IoT security issues based on NFV, SDN, and ML technologies. The figure displays the security component framework and interconnections, whereas Fig. 4 demonstrates the closed-loop automation phases, starting with detection and monitoring and ending with preventing threats. To ensure complete security, the system suggested integrating the enablers and countermeasures from the previous subsections. This framework enforces security policies beginning with the design and concluding with the application and maintenance. Two primary framework levels are shown in Fig. 3 (i.e., security orchestration and security enforcement layers). The two layers and their closed-loop automation intercommunications to detect and prevent attacks are discussed below.

1. *Security enforcement layer* Several VNFs implemented on many clouds, Physical Network Functions (PNFs), and edges facilitate interaction between IoT devices and end users. These network functions (PNFs and VNFs), end users, and IoT devices interact with each other over either a conventional or an SDN-based network. The research classifies attacks on the IoT as either *internal* or *external*. The *internal* attack is caused by compromised and malicious IoT devices, while the external attack is initiated from the end-user network and directed at the IoT domain. The *external* attack creates danger for the external network and/or other authorized IoT devices. Attacks would be primarily addressed at three levels: (1) IoT devices, via IoT controllers; (2) network, via SDN controllers; and (3) cloud, via an NFV orchestrator. By implementing VNF security and setting the interaction through SDN networking, the security framework features may be properly implemented within the IoT territory. The security enforcement plan was developed to match closely with ETSI and Open Networking Foundation (ONF) guidelines for NFV and SDN. As shown in Fig. 1, the security enforcement mechanisms consist of five separate logical blocks.
  *Management and control block* It analyzes the components required to manage NFV and SDN infrastructures. It uses SDN controllers and ETSI MANO stack modules for this. To implement efficient security functions, the SDN controllers and NFV orchestrator must work closely together as NFV is frequently used alongside SDN to alter programmatically the network based on policies and resources.
  *VNF block* Taking into consideration the VNFs that have been implemented across the virtualization infrastructure to implement various network-based security measures, the threat and protection measures required by the rules of security will be met with a focus on the delivery of sophisticated VNF security (e.g., IDS/IPS, virtual firewalls, etc.).
  *Infrastructure block* It includes every hardware component needed to construct an IaaS layer, including computers, storage devices, networks, and the software used to run them in a virtualized environment. In addition to the elements of the network that are in charge of transmitting traffic while adhering to the regulations that have been specified by the SDN controller, a set of security probes is included in this plane to gather data for use by the monitoring services.
  *Monitoring agents block* Its primary duty is reporting network activity and IoT actions to identify and prevent various types of attacks. In the proposed model, the detection technique may make use of either network patterns or IoT misbehavior. Using SDN-enabled traffic mirroring, every bit of data that is being sent over the network can be seen. The Security Orchestration Plane hosts an AI-based response agent that receives logs from the monitoring agents describing malicious transactions.
  *The IoT domain block* It refers to the interconnected system of cameras, sensors, appliances, and other physical objects that form the SDN. The proposed methodology considers the substantial risk these devices pose to data privacy and integrity, and it tries to enforce the security standards in this domain.
2. *Security orchestration layer* This layer has the task of setting up real-time rules of security depending on the current state of monitoring data and adjusting the policies dynamically based on their context. It is a novel part of the proposed framework that communicates with the security enforcement layer to request the necessary actions to be taken to enforce security regulations inside the IoT domain. Virtual security enablers must be created, configured, and monitored to deal with the present attack.
  Figure 2 is a diagrammatic representation of the major cooperation that happens among various framework components. This study proposes a feedback automation mechanism control system consisting of an oversight agent, an AI-based reaction agent, and an orchestrator for security. The latter protects against dangers by utilizing an NFV orchestrator, SDN controller, and IoT controller (see Figs. 3, 4).
  *AI-based reaction agent* This part orders the security orchestrator to perform predetermined measures in response to an incident. This block, as shown in Fig. 4, makes use of the information collected by the monitoring agent from IoT domains and the network. This part employs ML models that have been trained on network topologies and the actions of IoT devices to identify potential dangers. For the security orchestrator, these ML models will be able to prescribe the optimal template for policies of security. Figure 4 also shows how to identify security threats from observations of network patterns and/or IoT activities. The security orchestrator would then be informed of the discovered danger level (where every level from L1 to L5 belongs to a different predefined security policy). As shown in Fig. 4, we developed an AI-based reaction agent that uses seven ML techniques to recognize IoT-related attack activities and/or patterns in a network. These techniques are Random Forest, Decision Tree, Naive Bayes, Backpropagation NN, XGBoost, AdaBoost, and Ensemble RF-BPNN.
  *Security orchestrator* This part of closed-loop automation enforces the AI reaction agent’s security practices. It enforces IoT security regulations utilizing SDN and NFV with the control and management block. The security orchestrator instantiates, configures, and monitors virtual security devices, manipulates bad traffic through SDN, or directly controls IoT machines, like shutting off a hacked device.

We have addressed the IoT security threats using RF, NB, DT, NNs, XGBoost, AdaBoost, and Ensemble RF-BPNN, which involve leveraging ML algorithms to detect and mitigate potential risks. To highlight their effectiveness, we can compare some of these approaches to traditional security methods as follows:

1. RFs are an ensemble learning algorithm that combines multiple DTs to enhance accuracy and robustness. They applied to the proposed IoT security system as follows:
  - *Ensemble construction* RF consists of multiple DTs, each trained on a randomly selected subset of the training dataset. This randomness helps to reduce overfitting and increase generalization.
  - *Classification* When classifying new instances, each DT in the RF independently predicts the class. The last prediction depends on the majority vote or averaging of the individual tree predictions.
2. Decision trees (DTs) are a popular ML technique for classification and regression tasks. The proposed IoT security system uses a DT classifier to identify and address unique threats, and it works as follows:
  - *Feature selection* The first stage is to select relevant features from the IoT device data. These features can include network traffic patterns, device behavior, communication protocols, and more.
  - *Training* Using a labeled dataset, we train a DT classifier that contains instances of both normal and malicious behavior. The model learns to classify instances based on the selected features.
  - *Detection* Once trained, the DT can classify new instances as normal or malicious, depending on their feature values. If the DT classified an instance as malicious, it would take appropriate security measures, such as blocking network access or raising an alarm.
3. *Neural networks* NNs, particularly DL architectures, have gained significant popularity in various domains, including IoT security. Here’s how they can be used:
  - Multiple layers of interconnected nodes (neurons) form the architecture design of a neural network model. Each neuron applies a non-linear activation function to weighted inputs from the previous layer.
  - We train the neural network using a labeled dataset through a process known as backpropagation. To reduce the discrepancy between the expected and observed labels, we iteratively tweak the network’s biases and weights.
  - Prediction: Once trained, the neural network can classify new instances into different threat categories based on their input features.

- *Comparative analysis with traditional approaches* Compared to traditional security approaches, such as rule-based systems or signature-based detection, ML techniques offer several advantages. Traditional methods rely on predefined rules or patterns, which might not be able to adapt to rapidly evolving threats. In contrast, ML methods can learn from data and adapt their behavior accordingly. They can detect anomalies, identify new attack patterns, and improve over time as they encounter new threats. However, traditional approaches often provide better interpretability and explainability.

Rule-based systems explicitly define security rules, making it easier for security analysts to understand and verify their behavior. However, ML models, especially complicated ones like neural networks, are black boxes, making their decision-making process difficult to comprehend.

In conclusion, ML techniques like DTs, RFs, XGBoost, AdaBoost, and neural networks provide powerful tools for addressing unique IoT security threats. They offer improved accuracy, adaptability, and the ability to handle complex and evolving attack patterns. However, they may trade off some interpretability compared to traditional security approaches. The approach is selected based on the specific requirements of the IoT security system and the trade-offs between accuracy, interpretability, and computational requirements.

### Performance evaluation of the proposed model

The experimental methodology and analysis outcomes of the AI-based response agent are covered in this section. An AI-based response agent can identify potential threats by performing the following steps: (1) Evaluate network patterns. To identify various forms of network infiltration, the research presents a knowledge-based intrusion detection framework. (2) Examine the strange behaviors that have been seen in the IoT system. Here, attacks are uncovered through the investigation of strange actions taken by IoT devices. To appropriately categorize the degree of the attacks and select the right security solutions, the research has applied supervised learning algorithms. The AI-based reaction agent will employ many ML approaches, considering the appropriate inputs from the monitoring agents, to remove a specific attack.

- *Evaluating network patterns* Intrusion system evaluation is the first stage in evaluating the framework’s effectiveness.

Several publicly available datasets, including the UNSW_NB15, IoT-23, DARPA, KDD 99, NSL-KDD, DEFCON, and balanced BoTNeT-IoT-L01 datasets, were used to build the proposed system (see the datasets link (https://drive.google.com/drive/folders/1gjP-pQzFZsLh2QMsIa5GPhEh5etv9Jvc?usp=sharing)). These datasets contain information on IoT attacks in the form of (.csv) files. Table 1 shows the network traffic information from different IoT devices. Advantages of the NSL-KDD dataset compared with the initial KDD dataset: The train set does not contain duplicated data; therefore, classifiers are not biased toward more frequent records. BoTNeT-IoT-L01 is a recent dataset that consists of two Botnet assaults (Gafgyt and Mirai). Over a 10-s frame with a decay factor of (0.1), the mean, count, variance, radius, magnitude, correlation coefficient, and covariance were the seven statistical measures that were computed. The .csv file was used to extract four features: jitter, packet count, outbound packet size alone, and combined outbound and inbound packet size^37^. By computing three or more statistical measures for each of the four traits, a total of twenty-three features were obtained.

**Table 1:** BoTNet-IoT-L01 balanced dataset traffic’s type and counts.

Traffic	Class	Type	No. of records
Normal	–	–	555,932
Attacks	Mirai and Gafgyt	UDP	186,062
	Mirai	Scan	67,626
		Syn	62,917
		Ack	54,864
		UDP plain	44,624
	Gafgyt	TCP	73,405
		Combo	44,091
		Junk	22,343

Furthermore, this study used the widely recognized NSL-KDD dataset as a benchmark. It served as a benchmark for assessing intrusion detection systems in this research. It is a much better version of dataset KDD 99 (see Table 2). The NSL-KDD dataset has over 21 distinct attack types, which serve as the foundation for the application of our proposed IDS model, such as teardrop, satan, rootkit, buffer-overflow, smurf DDoS, pod-dos, and Neptune-dos. The NSL-KDD dataset is primarily composed of preprocessed network traffic data. These data provide a more precise representation of the network traffic that occurs at present. There are two distinct collections of data inside the dataset: a set for *testing* and a set for *training*. Comparatively, the set of testing has around 23,000 records, whereas the training set contains approximately 125,000 records. Each entry in the dataset corresponds to a network connection and contains a set of 41 features, including the IP addresses of the source and destination, protocols, flags, and a label indicating whether the connection is normal or abnormal (anomalous). Each sample in the dataset corresponds to certain attacks as follows: DoS attacks, remote-to-local (R2L) attacks, user-to-root (U2R) attacks, and probing attacks^38^. There are many implementation tools available for analyzing IoT attack datasets, such as Wireshark, Snort, Zeek (formerly Bro), Jupyter Notebook, Python, and Weka. In this work, the researchers used Python programming and Weka data mining tools for ML and data analysis processing.

**Table 2:** Public used datasets for IoT detection system.

Dataset	No. of features	No. of instances	Name of attacks	Separate train- test set
BoTNet-IoT-L01	23	1,111,864	UDP, Scan, Syn, Ack, TCP, UDP plain, Combo, and Junk	Yes
NSL-KDD	42	148,517	DoS, Probe, R2L,and U2R	Yes
KDD99	42	4,886,431	DoS, Probe, R2L,and U2R	Yes
UNSW-NB15	49	1,540,044	DoS, Fuzzers, Backdoors, Worms, Reconnaissance, Analysis, Exploits, Generic, and Shellcode	Yes

The proposed tools include a large collection of ML algorithms for classification, regression, clustering, and association rule mining, such as RF, NB, DT, NNs, XGBoost, AdaBoost, and Ensemble RF-BPNN, as well as tools for model evaluation and selection, including cross-validation and ROC analysis.

Certain ML algorithms are incapable of learning due to the wide range of features present in nature. The modeling process becomes more challenging when a feature is continuous. Hence, before constructing classification patterns, preprocessing is fundamental to optimize prediction accuracy. Specifically, a discretization technique is used to overcome this restriction. When applied to a continuous variable, the discretization data mining approach seeks to minimize the number of possible values by categorizing them into intervals. Two different kinds of discretization are discussed in the literature: (1) *static variable discretization*, in which variables are partitioned separately, and (2) *dynamic variable discretization,* in which all features are discretized concurrently^39^. The research discretized the attacks and then categorized them such that the research was left with only the most common types (UDP, Junk, Ack, and UDP plain from the balanced BoTNet-IoT-L01 dataset and DDoS, Probe, U2R, and R2L from NSL-KDD).

1. *Metrics for comparing performance* Choosing measures that can indicate the strength of an IDS is a major problem when evaluating an IDS. An IDS’s performance goes well beyond its classification results alone. Cost Per Example (CPE), precision, detection rate, and model accuracy are utilized to evaluate the effectiveness of the proposed system. When evaluating outcomes, the following metrics should be used in conjunction with one another^40^.
  1 $$CPE = \frac{1}{N} \sum_{i=1}^{5}\sum\limits_{j=1}^{5}CM \left(i,j\right)+C\left(i,j\right).$$
  Equation (1) indicates Cost-Sensitive Classification (CSC) or CPE, *where N* is the total number of samples, CM refers to the classification’s Confusion Matrix algorithm, and C is the Cost Matrix (see Table 3)^41^.
2. *Input data cleaning, feature extraction, and classification* The research proposes a first method, which involves preparing the entire dataset and then categorizing it using a variety of techniques (Hoeffding Tree, RF, Bayes Net, and J48) as shown in Fig. 6. Next, the research chooses the best classifier (algorithm) that generates a preferred accuracy (see Table 4 for the BoTNet-IoT-L01 dataset and Table 5 for the NSL-KDD dataset).
3. *Backpropagation approach* To investigate the multilayer neural net approach, the research utilized the capabilities of a backpropagation technique for learning. The research employed a multilayer neural network with three layers. The initial layer had 41 inputs, representing the features of the dataset. The final layer encompassed the classification responses, namely, U2L, U2R, Probe, DoS, and Normal. An extra hidden layer was incorporated to facilitate the learning process. This method uses 100 neurons and a single hidden layer. Experience has shown that the alternative hidden layer and neuron counts did not increase the mean squared error (MSE) (see Table 6).
4. *Distributed classification module* This module introduces a distributed categorization system in which the various types of attacks (DDoS, U2R, R2L, and Probe; UDP, UDP plain, Ack, and Junk) are all assigned to the Ensembled RF-BPNN algorithm. Finally, the AdaBoost method is used to combine the resulting models (see Table 7).

**Table 3:** The cost matrix for analyzing the NSL-KDD dataset.

	U2L	U2R	Probe	DoS	Normal
U2L	0	1	1	1	4
U2R	1	0	2	2	2
Probe	2	1	0	2	1
DoS	1	2	2	0	2
Normal	2	2	1	2	0

**Table 4:** Performance assessment of different ML methods with a balanced BoTNet-IoT-L01 dataset.

Algorithm	Accuracy (%)	Sensitivity (%)	Specificity (%)	Training time (s)	Testing time (s)
Random forest	98.4	95.5	98.8	192.7	92.08
Naive Bayes	78.0	76.2	76.8	214.55	82.18
Decision tree	95.8	95.6	98.1	219.61	82.13
Backpropagation NN	95.3	94.6	94.2	258.91	88.20
Ensembled RF-BPNN	99.2	97.6	96.2	230.4	86.66

**Table 5:** The precision rate of used ML algorithms for each attack from NSL-KDD.

	Hoeffding tree	RF	Bayes Net	J48
U2L	75.8%	99.4%	65.7%	98.1%
U2R	77.2%	83.4%	65.5%	73.8%
Probe	98.2%	99.8%	85.3.%	99.5%
DoS	99.4%	100%	99.8%	99.9%
Normal	96.4%	99.6%	97.8%	99.8%
Time	4.6	73.8	5.7	43.6
Precision	98.9%	99.8%	98.7%	99.8%
FTR	2.3%	0.1%	1.7%	0.3%
Detection rate	98.8%	99.8%	97.5%	99.8%
CPE	7.5%	0.2%	6.8%	0.5%

**Table 6:** Backpropagation evaluation on the NSL-KDD dataset.

	U2L	U2R	Probe	DoS	Normal	Model (%)
Precision	89.4%	77.7%	99.1%	99.1%	98.8%	98.9
FTR	0.1%	0%	0.1%	0%	0.2%	1.0
Detection rate	78.4%	79.6%	98.7%	99.1%	98.9%	98.9
CPE	–	–	–	–	–	2.8

**Table 7:** The evaluation metrics of the AdaBoost technique.

	U2L	U2R	Probe	DoS	Normal	Model (%)
Precision	89.4%	87.7%	99.9%	100%	99.8%	99.9
FTR	0%	0.1%	0.1%	0%	0.2%	0.1
Detection rate	87.4%	79.9%	99.7%	100%	99.8%	99.9
CPE	–	–	–	–	–	0.3

## Result evaluation and discussion

The findings reported in Table 5 demonstrate both the accuracy rate and precision of the RF technique. Unfortunately, the results are not promising for either U2R or U2L attacks. There is a low misclassification rate (or CPE) and high accuracy when using J48 to identify attacks. When it comes to the accuracy required for U2R strikes, however, J48 falls short. Despite its consistent performance, the Hoeffding tree method has a low accuracy for U2R threats. Although it has a strong model accuracy, the Bayes Net method provides the lowest results, failing to identify the vast majority of U2R threats. As can be seen from Table 6, the backpropagation process is generally as precise as its predecessors, if not somewhat more so. However, misclassification comes with a significant processing time penalty. AdaBoost, CPE, and detection rate produced a better detection accuracy model as shown in Table 7.

### The performance of ML algorithms used in the proposed system

**Figure 5:**
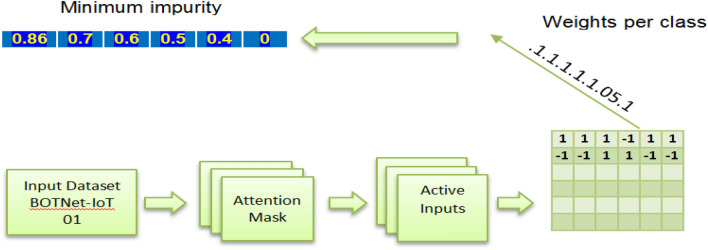
Architectural flow graph of the proposed RF with backpropagation NN (RF-BPNN).

A classification algorithm for IoT detection based on ensembles of backpropagation neural networks is trained on the BoTNet-IoT-L01 dataset (see Table 8). The novelty of the algorithm stems from the methodology employed for combining outputs of the backpropagation neural network ensembles. The backpropagation neural network Oracle 8i database tool is utilized to combine the ensemble outputs. As Fig. 5 shows, the neural network backpropagation Oracle is constructed with an RF algorithm that produces high classification accuracy and low classification error (see Table 4). The thresholds are not learned all at once in the RF model but rather hierarchically. The decrease in impurity will be enforced one directionally from the starting to the finishing index of the symbolic path; however, the research learned them simultaneously. The idea of hierarchical node splits will be represented by this one-directional impurity reduction. To do this, firstly, the research breaks up each node in the symbolic path into some votes for each class. Secondly, the research computes the impurity based on those votes. The third step is to gradually lower it by a certain amount using the Softmax activation method. Our proposed algorithm uses margin ranking loss as its objective function. It is important to maintain a minimum margin disparity between the intended result and the actual one. The margin difference is the ‘reduction in impurity’. The target is output shifted by one index to the right and the impurity at first split is initialized by the impurity of the batch (see Fig. 5).

**Table 8:** Evolution parameters for RF-backpropagation NN.

Parameters	Values
Epochs no.	500
Layers count in the NN	{1–4}
Units count in the NN	{100–200}
Percent of noise applied c	25%
Learning percent	0.005
Classifier (output layer)	RF

**Figure 6:**
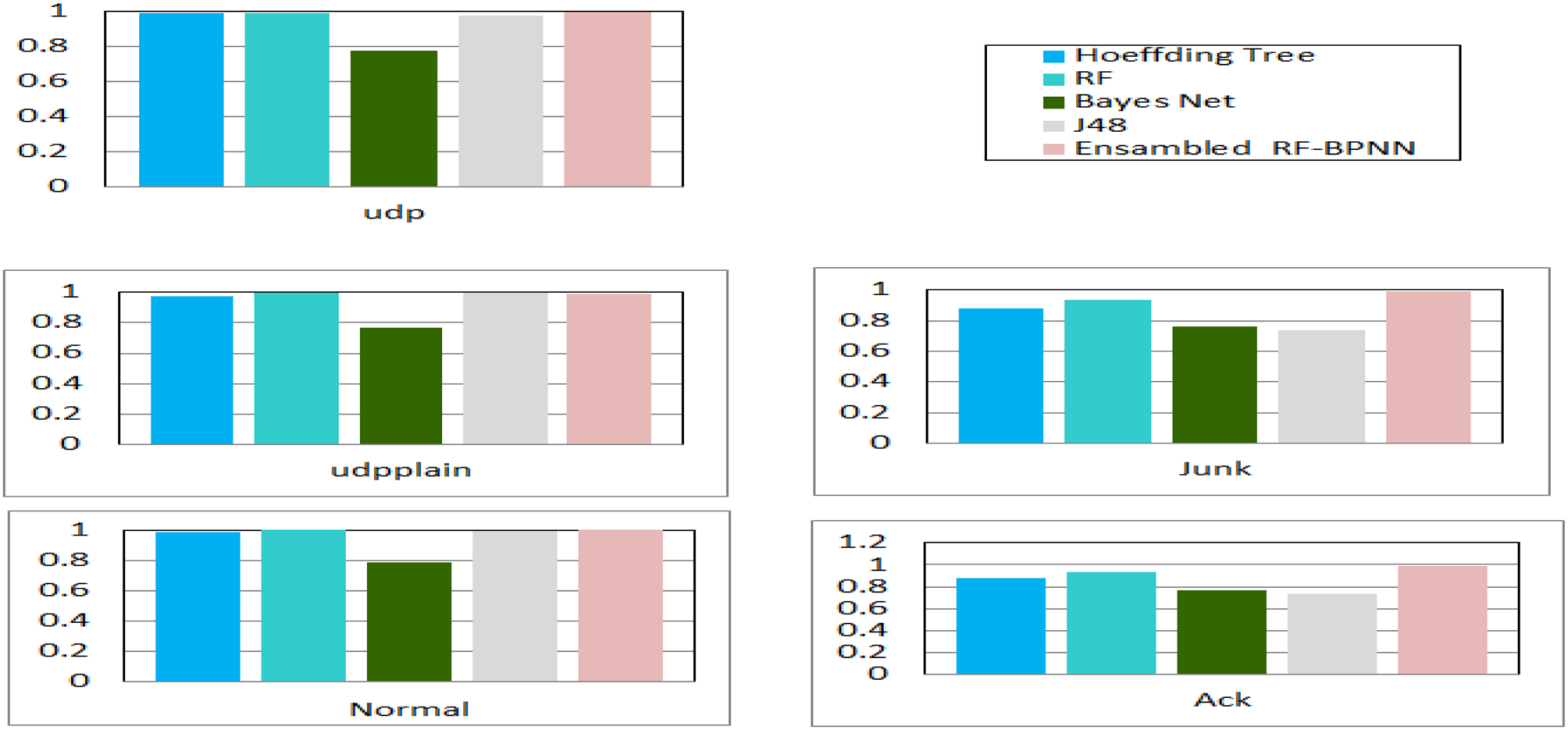
RF-BPNN accuracy evaluation for each attack type in the balanced BoTNet-IoT-L01 dataset.

When employing the AdaBoost classifier as a detection model, the research was limited to considering a single window size. Therefore, the research has successfully decreased the number of attributes in the BoTNeT-IoT-L01 dataset from 115 to 23. This significant decrease in the dimensionality of the dataset results in a significant acceleration of the detection process. Speaking of the BotNet-IoT dataset, the research discovered that just a small number of parameters have an important role in our system’s overall performance, and time windows of 10 s performed marginally better than those of shorter duration (see Fig. 6). Additionally, the research discovered that traffic heterogeneity greatly impacted RF classifier performance. However, when compared to the other classification algorithms, AdaBoost and RF-BPNN had the greatest and most stable results (see Table 7).

**Figure 7:**
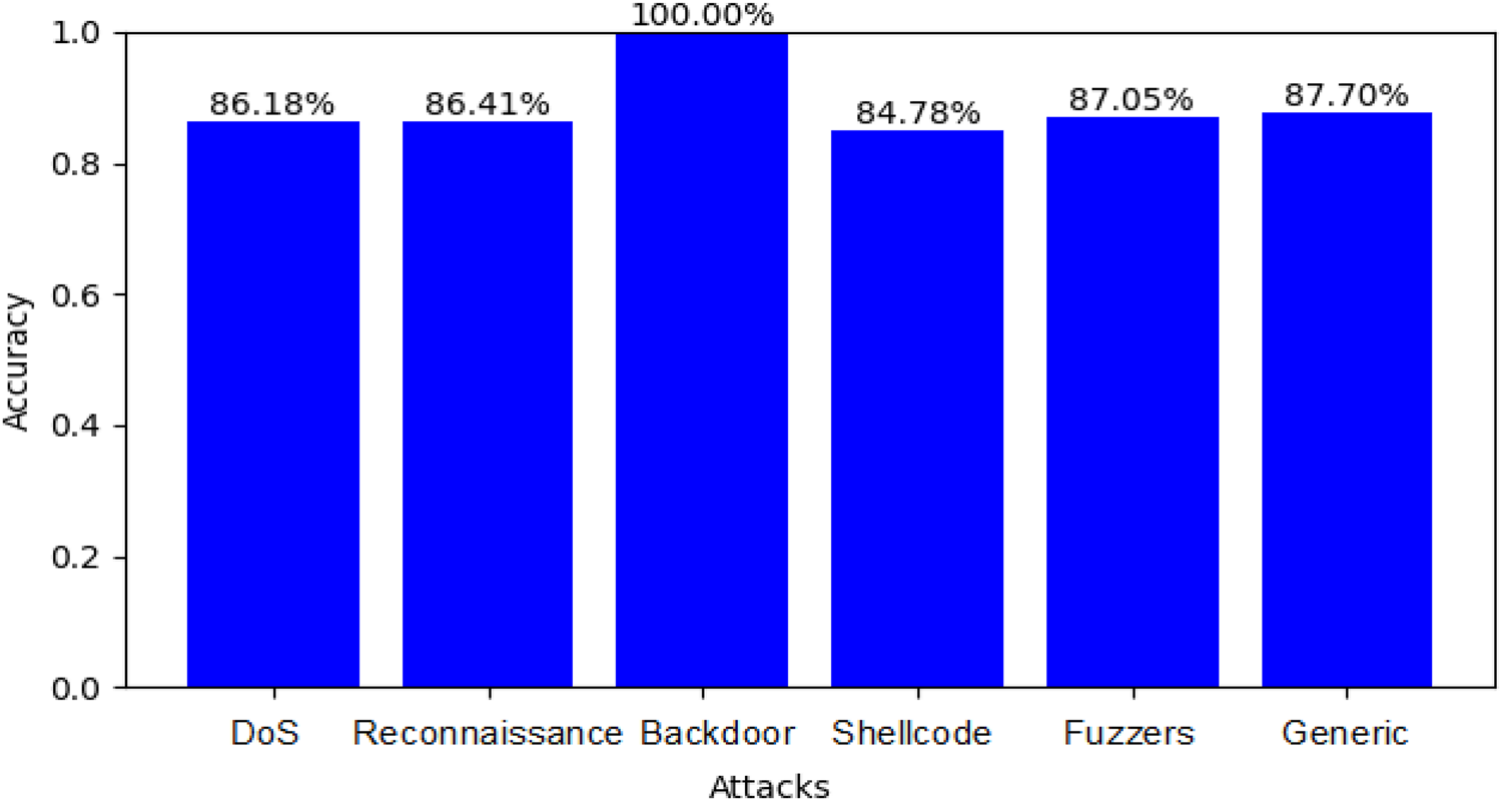
The accuracy for detecting some attacks in the UNSW_NB15 dataset, using RF Classifier.

Figure 7 shows the accuracy for detecting *DoS*, *Fuzzers*, *Gene*ric, *Backdoor,* and *Exploit* attacks in the UNSW_NB15 dataset using the RF classifier and SMOTE (where “*label”* refers to the target variable and *“attack_cat*” refers to the attack types).

Different experiments determine the system’s performance. Examining and validating each stage using the supplied classifiers is necessary to confirm the experimental results. Whether the classifier can discriminate across feature categories is also crucial. Accuracy, specificity, precision, recall, F1-score, and AUC measure the model’s performance and indicate the correctness of the system. Such measurements are based on the T_P_, F_P_, T_N_, and F_N_, as shown in Eqs. (2) to (6):

2 $$Accuracy =\frac{{{T}_{P}+T}_{N} }{{{T}_{N}+T}_{P}+{{F}_{N}+F}_{P}},$$

3 $$Precision =\frac{{T}_{P}}{{T}_{P}+{F}_{P}},$$

4 $$Specificity =\frac{{T}_{N} }{{{T}_{N} + F}_{P}},$$

5 $$Recall = \frac{{T}_{P}}{{T}_{P}+{F}_{N}},$$

6 $$F1-score = \frac{2 \times Recall \times Percision }{Recall + Percision}.$$

We use the following terms to describe the classification errors: true positive (TP) for attack instances, true negative (TN) for normal cases, false positive (FP) for incorrectly classified normal instances, and false negative (FN) for incorrectly classified attack instances.

Thus, the accuracy formula evaluates the classifier’s capacity to accurately categorize both positive and negative instances; precision denotes the classifier’s ability to avoid incorrectly labeling positive instances as negative, and specificity denotes its capacity to avoid incorrectly labeling negative instances as positive. In machine learning, recall is the rate at which a classifier can identify positive examples, whereas the F1-score is the weighted average of accuracy and recall.

**Figure 8:**
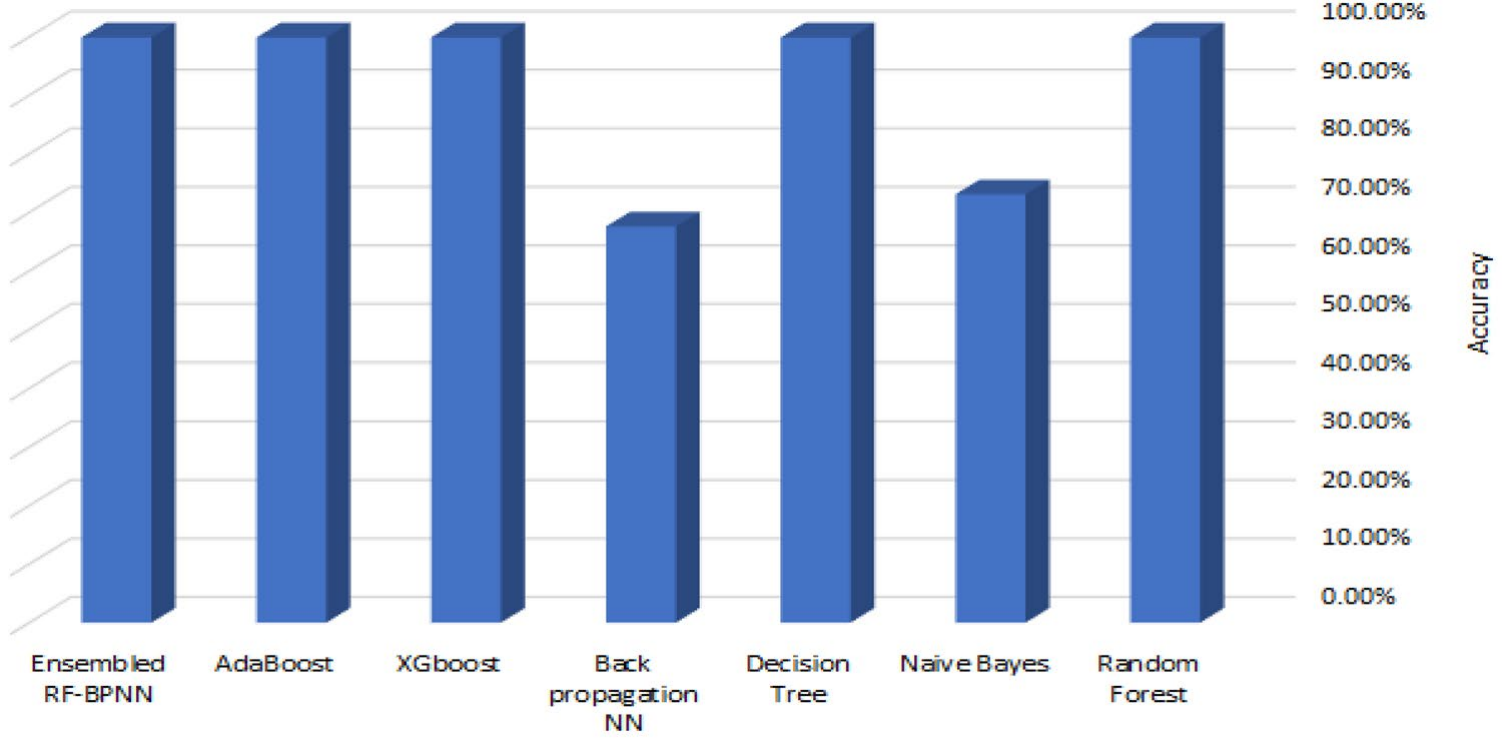
The accuracy of 7 ML algorithms using the UNSW-NB15 dataset and SMOTE.

Table 9 shows the performance of seven machine learning classifiers using the Synthetic Minority Oversampling Technique (SMOTE) on the UNSW_NB15 dataset. As you can see in Fig. 8, the RF, XGBoost, AdaBoost, and Ensembled RF-BPNN classifiers did the best overall. They achieved an accuracy of 99.9%, an AUC of 1, and an F1 score of 99.9%. The Naive Bayes classifier, on the other hand, obtained the *minimum* accuracy and F1 score.

**Table 9:** Performance metrics for 7 ML algorithms using the UNSW-NB15 dataset and SMOTE.

Ser.	Classifier	Accuracy (%)	Precision (%)	Specificity (%)	Recall (%)	F1-score (%)	AUC
1	Random forest	99.9	99.9	99.8	99.9	99.9	1.0
2	Naive Bayes	73.1	73.8	65.6	79.0	76.3	0.8
3	Decision Tree	99.9	100.0	100.0	99.1	99.5	0.8
4	Back propagation NN	67.6	66.7	49.9	82.0	73.6	0.6
5	XGBoost	99.9	100.0	100.0	99.1	99.9	1.0
6	AdaBoost	99.9	99.9.0	99.9	99.9	99.9	1.0
7	Ensembled RF-BPNN	99.9	99.9	99.8	99.9	99.9	1.0

### Integration with existing IoT security frameworks and standards

The proposed model can integrate with existing IoT security frameworks and standards as follows:

1. *Integration with IoT security frameworks* The ML-based model can integrate with IoT security frameworks by aligning its functionalities with their security objectives and guidelines. For example:
  - The proposed model can integrate with existing authentication mechanisms recommended by IoT security frameworks, such as digital certificates or secure bootstrapping protocols. It can enhance device authentication by analyzing device behavior patterns and detecting anomalies that may indicate unauthorized access or compromised devices.
  - To align with data privacy requirements, the model can utilize encryption techniques and privacy-preserving algorithms recommended by the IoT security frameworks. It provides a guarantee of secure transmission and storage of data, protecting confidential information against illegal access.
  - The proposed model can integrate with existing access control mechanisms defined by IoT security frameworks. It can augment access control by providing intelligent decision-making capabilities based on historical data, user behavior analysis, or contextual information. This aids in assessing access requests and preventing unauthorized access to IoT resources.
2. *Integration with IoT security standards* The ML-based model can comply with IoT security standards by incorporating the required security controls and practices. For example:
  - The proposed model can align with ISO/IEC 27000 standards by implementing appropriate security controls for risk assessment, incident management, and data protection. It can follow the standards’ guidelines to ensure that the necessary security measures are in place.
  - The model can follow the NIST framework to enhance its threat detection and incident response capabilities.
3. *Interoperability in IoT ecosystems* By adhering to standard IoT protocols, data formats, and metadata standards, the ML-based model can ensure interoperability. For example:
  - The ML model can communicate with IoT devices and gateways using standard IoT protocols such as MQTT or CoAP, ensuring compatibility and interoperability across different devices and platforms.
  - The ML model can use commonly used data formats, such as JSON, or semantic data models, such as the Semantic Sensor Network (SSN) ontology, to facilitate seamless data sharing and interoperability with other components within the IoT ecosystem.

By integrating with existing IoT security frameworks and standards, the proposed model can enhance its adaptability and compatibility within IoT ecosystems. This integration allows the model to complement and enhance the existing security infrastructure, contributing to improved IoT security outcomes.

### Comparisons with related systems

Table 10 highlights the proposed model’s performance outcomes by comparing it to previous systems. This study looked at existing literature and compared it to others based on standards, like the false positive rate (FPR), CPE, accuracy, and detection rate^38–47^. Through several experiments, the proposed system achieved the best evaluation metrics for accuracy, precision, detection rate, CPE, and lowest time complexity compared with previous solutions, as shown in Tables 10 and 11.

**Table 10:** Comparing the results with earlier studies.

	Accuracy (%)	Detection rate	FPR	Training time
Ambusaidi et al.^38^	92.3	92.3%	0.41	–
Moustafa et al.^39^	97.8	97.8%	2.5%	–
Tsai et al.^40^	96.9	97.8%	2.5%	–
Alom et al.^41^	97.5	–	–	3.2 s
Yin et al.^42^	99.5	97.1%	3.6%	5516 s
Tang et al.^43^	75.8	75.0%	15%	–
Ludwig^44^	92.5	98.0%	14.7%	–
Al-Hawawreh et al.^45^	98.6	99.0%	1.8%	398 s
Shone et al.^46^	98.0	71.0%	-	–
Subba et al.^47^	97.6	97.3%	-	–
The proposed app (experiment #1)	99.1	97.3%	0.2%	425.5
The proposed app (experiment #2)	99.5	98.6%	0.7%	230.4
The proposed app (experiment #3)	99.9	99.8%	0.1%	192.7

**Table 11:** Time complexity of training and testing for the ML algorithms used.

Algorithm	Training (time complexity)	Testing (Time complexity)	Auxiliary space	Notes
RF	O(n log(n)d n_tree_)	O(d n_tree_)	O(p n_tree_)	
Bayes Net	O(n*m)	O(c*m)	O(c*m)	
Decision Tree (Hoeffding Tree, J48)	O(n log(n)d)	O(d)	O(p)	
Backpropagation NN	O(n ∗ t ∗ (ij + jk + kl)),	O(nk)	O(nk)	n: number of epochs

### Privacy concerns and data bias

The authors of this work have incorporated essential steps into the development and deployment of the proposed ML-based security model to effectively address privacy concerns and data bias, as well as ensure the technology’s ethical and responsible use within the IoT system.

1. The authors conducted a *privacy impact assessment* to determine if the proposed ML-based security model has any privacy issues or concerns.
2. To mitigate privacy concerns, the study implemented *privacy-enhancing techniques*. This process included data anonymization, encryption, differential privacy, or federated learning, which allows for training the proposed ML model without sharing raw data.
3. The study *minimized* the amount of personally identifiable information (PII) gathered and stored to reduce privacy risks. During the requirements engineering phase, we only collected the necessary data for the proposed machine learning-based security model, ensuring its safe storage and disposal when no longer required.
4. We implemented regular monitoring of the proposed ML model for potential biases in data and outcomes. Implementing a *bias detection* process is critical for identifying discriminatory patterns. We can take steps to *mitigate detected biases*, which may include adjusting training data, diversifying datasets, or utilizing bias correction algorithms.
5. Regularly monitor the proposed ML-based security model performance, including privacy aspects, and update it as needed to address emerging privacy concerns, mitigate biases, and ensure ongoing compliance with ethical standards.

## Conclusions and upcoming work

This research introduces a new proposed ML-based security model to address the vulnerabilities in IoT systems. We designed the proposed model to autonomously handle the growing number of security problems associated with the IoT domain. This study analyzed the state-of-the-art security measures, intelligent solutions, and vulnerabilities in smart systems built on the IoT that make use of ML as a key technology for improving IoT security. The study illustrated the benefits and limitations of applying ML in an IoT environment and proposed a security model based on ML that can automatically address the rising concerns about high security in the IoT domain. The suggested method performs better in terms of accuracy and execution time than existing ML algorithms, which makes it a viable option for improving the security of IoT systems. This research evaluates the intrusion detection system using the BoTNet-IoT-L01 dataset. The research applied our proposed IDS model to a dataset that included more than 23 types of attacks. This study also utilized the NSL-KDD dataset to evaluate the intrusion detection mechanism and evaluated the proposed model in a real-world smart building environment. The presented ML-based model is found to have a good accuracy rate of 99.9% compared with previous research for improving IoT systems’ security. This paper’s contribution is the development of a novel ML-based security model that can improve the efficiency of cybersecurity systems and IoT infrastructure. The proposed model can keep threat knowledge databases up to date, analyze network traffic, and protect IoT systems from newly detected attacks by drawing on prior knowledge of cyber threats. This study presents a promising ML-based security approach to enhance IoT system security. However, future work and improvements remain possible. Expanding the dataset for the intrusion detection system evaluation could be one area of improvement. While the BoTNet-IoT-L01 and NSL-KDD datasets used in this study are comprehensive, they may not cover all possible types of attacks that could occur in an IoT environment. Therefore, our future research could focus on collecting and analyzing more diverse datasets to increase the performance of the proposed model. Furthermore, optimizing the proposed model’s execution time is crucial for real-world applications. Also, we could integrate the proposed model with other security solutions to create a more comprehensive and robust security system for IoT devices. Overall, the development of this novel ML-based security model is a significant contribution to the literature on ML security models and IoT security, and further work and improvements will continue to advance the field. Finally, the security analyst treats the AI-based IDS as a black box due to its inability to explain the decision-making process^48^. In our future work, we will expand our research by integrating blockchain-based AKA mechanisms with explainable artificial intelligence (XAI) to secure smart city-based consumer applications^49^. On the other hand, we can use the Shapley Additive Explanations (SHAP) mechanism to explain and interpret the prominent features that are most influential in the decision^50^.

## Data Availability

The corresponding author can provide the datasets used and/or analyzed in this work upon reasonable request.

## References

[1] Sharma A, Singh PK, Kumar Y (2020). An integrated fire detection system using IoT and image processing technique for smart cities. Sustain. Cities Soc..

[2] Sinan K (2020). SDG-11: Sustainable Cities and Communities. Emerging Technologies, Sustainable Development Goals Series.

[3] Hussain F, Hussain R, Hassan SA, Hossain E (2020). Machine learning in IoT security: Current solutions and future challenges. IEEE Commun. Surv. Tutor..

[4] Bharati S, Mondal MRH, Podder P, Prasath VB (2022). Federated learning: Applications, challenges and future directions. Int. J. Hybrid Intell. Syst..

[5] Shafiq M, Tian Z, Bashir AK, Du X, Guizani M (2020). Corrauc: A malicious BOT-IOT traffic detection method in IoT network using machine learning techniques. IEEE Internet Things J..

[6] Omolara AE, Alabdulatif A, Abiodun OI, Alawida M, Alabdulatif A, Alshoura WH, Arshad H (2022). The Internet of Things security: A survey encompassing unexplored areas and new insights. Comput. Secur..

[7] Bharati S, Podder P, Mondal MRH, Paul PK, Hassanien AE, Khamparia A, Gupta D, Shankar K, Slowik A (2021). Applications and challenges of cloud integrated IoMT. Cognitive Internet of Medical Things for Smart Healthcare.

[8] Özalp, A. N. *et al*. Layer-based examination of cyber-attacks in IoT. In *2022 International Congress on Human-Computer Interaction, Optimization and Robotic Applications (HORA)* (IEEE, 2022).

[9] Altunay HC, Albayrak Z (2023). A hybrid CNN+ LSTM—Based intrusion detection system for industrial IoT networks. Eng. Sci. Technol. Int. J..

[10] Abbas Y, Ali D, Gautam S, Hadis K, Reza MP (2024). Hybrid privacy preserving federated learning against irregular users in next-generation Internet of Things. J. Syst. Archit..

[11] Abbas Y, Ali D, Gautam S (2023). AP2FL: Auditable privacy-preserving federated learning framework for electronics in healthcare. IEEE Trans. Consumer Electron..

[12] Danyal N, Abbas Y, Ali D, Gautam S (2024). Federated quantum-based privacy-preserving threat detection model for consumer Internet of Things. IEEE Trans. Consumer Electron..

[13] Sanaz, N., Behrouz, Z., Abbas, Y. & Ali, D. Steeleye: An application-layer attack detection and attribution model in industrial control systems using semi-deep learning. In *2021 18th International Conference on Privacy, Security and Trust (PST), IEEE Xplore* (2021).

[14] Abbas Y, Ali D, Reza MP, Gautam S, Hadis K (2023). Secure intelligent fuzzy blockchain framework: Effective threat detection in IoT networks. Comput. Ind..

[15] Gopi KJ, Abbas Y, Reza MP, Seyedamin P (2023). Exploring privacy measurement in federated learning. J. Supercomput..

[16] Otoum, Y. & Nayak, A. On securing IoT from deep learning perspective. In *Proc. 2020 IEEE Symposium on Computers and Communications (ISCC)* 1–7 (2020).

[17] Butun I, Sterberg PO, Song H (2020). Security of the Internet of Things: Vulnerabilities, attacks, and countermeasures. IEEE Commun. Surv. Tutor..

[18] Tahsien SM, Karimipour H, Spachos P (2020). Machine learning based solutions for security of Internet of Things (IoT): A survey. J. Netw. Comput. Appl..

[19] Abiodun OI, Abiodun EO, Alawida M, Alkhawaldeh RS, Arshad H (2021). A review on the security of the Internet of Things: Challenges and solutions. Wirel. Person. Commun..

[20] Podder P, Mondal MRH, Bharati S, Paul PK (2020). Review on the security threats of Internet of Things. Int. J. Comput. Appl..

[21] Hamad ZJ, Askar S (2021). Machine learning powered IoT for smart applications. Int. J. Sci. Bus..

[22] Xu H, Przystupa K, Fang C, Marciniak A, Kochan O, Beshley M (2020). A combination strategy of feature selection based on an integrated optimization algorithm and weighted K-nearest neighbor to improve the performance of network intrusion detection. Electronics.

[23] Bharati S, Mondal MRH, Bharati S, Mondal MRH (2021). Computational intelligence for managing pandemics. 12 Applications and Challenges of AI-Driven IoHT for Combating Pandemics: A Review.

[24] Robel MRA, Bharati S, Podder P, Mondal MRH, Gupta D, Khamparia A (2020). IoT driven healthcare monitoring system. Fog, Edge, and Pervasive Computing in Intelligent IoT Driven Applications.

[25] Podder P, Mondal MRH, Kamruzzaman J, Elngar AA, Chowdhury R, Elhoseny M, Balas VE (2022). Iris feature extraction using three-level Haar wavelet transform and modified local binary pattern. Applications of Computational Intelligence in Multi-Disciplinary Research.

[26] Chandavarkar, B. R. Hardcoded credentials and insecure data transfer in IoT: National and international status. In *Proc. 2020 11th International Conference on Computing, Communication and Networking Technologies (ICCCNT)* 1–7 (2020).

[27] Ferrara P, Mandal AK, Cortesi A, Spoto F (2021). Static analysis for discovering IoT vulnerabilities. Int. J. Softw. Tools Technol. Transf..

[28] Yu Y, Guo L, Liu S, Zheng J, Wang H (2020). Privacy protection scheme based on CP-ABE in crowdsourcing-IoT for Smart Ocean. IEEE Internet Things J..

[29] Xiong J, Ma R, Chen L, Tian Y, Li Q, Liu X, Yao Z (2020). A personalized privacy protection framework for mobile crowdsensing in IIoT. IEEE Trans. Ind. Inform..

[30] Jiang X, Lora M, Chattopadhyay S (2020). An experimental analysis of security vulnerabilities in industrial IoT devices. ACM Trans. Internet Technol..

[31] Visoottiviseth, V., Sakarin, P., Thongwilai, J. & Choobanjong T. Signature-based and behavior-based attack detection with machine learning for home IoT devices. In *Proc. 2020 IEEE Region 10 Conference (TENCON 2020)* 829–834 (2020).

[32] Turk Z, Soto BGD, Mantha BRK, Maciel A, Georgescu A (2022). A systemic framework for addressing cybersecurity in construction. Autom. Construct..

[33] Al Hayajneh A, Bhuiyan NZA, McAndrew I (2020). Improving internet of things (IoT) security with software defined networking (SDN). Computers.

[34] Hussain F, Hassan SA, Hussain R, Hossain E (2020). Machine learning for resource management in cellular and IoT networks: Potentials, current solutions, and open challenges. IEEE Commun. Surv. Tutor..

[35] IoT Dataset for Intrusion Detection Systems (IDS). https://www.kaggle.com/azalhowaide/iot-dataset-for-intrusion-detection-systems-ids (2023).

[36] Nawir, M., Amir, A., Yaakob, N. & Lynn, O. B. Internet of Things (IoT): Taxonomy of security attacks. In *Proc. 3rd International Conference in Electronic Design (ICED)* 321–326 (2016).

[37] Herzberg, B., Bekerman, D. & Zeifman, I. Breaking down mirai: An IoT DDoS botnet analysis. Incapsula Blog, Bots and DDoS, Security, (2016).

[38] Ambusaidi MA, He X, Nanda P, Tan Z (2016). Building an intrusion detection system using a filter-based feature selection algorithm. IEEE Trans. Comput..

[39] Moustafa N, Creech G, Slay J, Moustafa N, Creech G, Slay J (2017). Big data analytics for intrusion detection system: Statistical decision-making using finite Dirichlet mixture models. Data Analytics and Decision Support for Cybersecurity.

[40] Tsai CF, Lin CY (2010). A triangle area based nearest neighbors approach to intrusion detection. Pattern Recogn..

[41] Alom, M. Z., Bontupalli, V. & Taha, T. M. Intrusion detection using deep belief networks. In *Proc. IEEE National Aerospace and Electronics Conference (NAECON)* 339–344 (2015).

[42] Yin C, Zhu Y, Fei J, He X (2017). A deep learning approach for intrusion detection using recurrent neural networks. IEEE Access.

[43] Tang, T. A., Mhamdi, L., McLernon, D., Zaidi, S. A. R. & Ghogho, M. Deep learning approach for network intrusion detection in software defined networking. In *Proc. 2016 International Conference on Wireless Networks and Mobile Communications (WINCOM)* 258–263 (2016).

[44] Ludwig, S. A. Intrusion detection of multiple attack classes using a deep neural net ensemble. In *Proc. 2017 IEEE Symposium Series on Computational Intelligence (SSCI)* 1–7 (2017).

[45] Al-Hawawreh M, Moustafa N, Sitnikova E (2018). Identification of malicious activities in industrial Internet of Things based on deep learning models. J. Inf. Secur. Appl..

[46] Shone N, Ngoc TN, Phai VD, Shi Q (2018). Deep learning approach to network intrusion detection. IEEE Trans. Emerg. Top. Comput. Intell..

[47] Subba, B., Biswas, S. & Karmakar, S. Enhancing performance of anomaly-based intrusion detection systems through dimensionality reduction using principal component analysis. In *Proc. 2016 IEEE International Conference on Advanced Networks and Telecommunications Systems (ANTS)* 1–6 (2016).

[48] Kumar R, Javeed D, Aljuhani A, Jolfaei A, Kumar P, Islam AKMN (2024). Blockchain-based authentication and explainable AI for securing consumer IoT applications. IEEE Trans. Consumer Electron..

[49] Javeed D, Gao T, Kumar P, Jolfaei A (2024). An explainable and resilient intrusion detection system for industry 5.0. IEEE Trans. Consumer Electron..

[50] Kumar R (2024). Digital twins-enabled zero touch network: A smart contract and explainable AI integrated cybersecurity framework. Future Gener. Comput. Syst..

